# Comparison of direct anterior versus posterolateral approach total hip arthroplasty for developmental dysplasia of the hip: A clinical effectiveness retrospective study

**DOI:** 10.1097/MD.0000000000042024

**Published:** 2025-04-04

**Authors:** Wuyuanhao Lin

**Affiliations:** a Orthopaedics Department, The First Affiliated Hospital of Nanchang University, Nanchang, Jiangxi Province, China.

**Keywords:** developmental dysplasia of the hip, direct anterior approach, posterolateral approach, total hip arthroplasty

## Abstract

The aim of this study was to evaluate the clinical efficacy of 2 approaches to total hip arthroplasty—the direct anterior approach and the posterolateral approach—in the treatment of developmental dysplasia of the hip. A total of 201 patients who were hospitalized between 2018 and 2023 for this condition were included in the study. Of the total number of patients, 100 underwent the procedure via the direct anterior approach (study group), whereas 101 underwent total hip arthroplasty via the posterolateral approach (control group). A range of clinical and patient data was gathered, including the following: age, gender, body mass index, disease classification, symptom history, intraoperative blood loss, blood transfusion volume, incision length, operation time, hospital stay, visual analog scale score, Harris score, Barthel index, postoperative complications, follow-up time, leg length discrepancy, and femur offset difference. The lack of statistically significant variations in age, gender, body mass index, and symptom history among the 2 patient groups suggests that they were comparable. Nevertheless, notable disparities were observed between the groups with regard to the length of the surgical incision (*P* < .001) and intraoperative blood loss (*P* < .001). Significant differences (*P* < .001) were observed in the visual analog scale scores of the patients in the study group at 1 day (6.71 ± 0.46), 3 days (5.71 ± 0.46), and 1 week (0.96 ± 0.20) after surgery, in comparison with the control group (7.46 ± 0.51, 6.35 ± 0.49, 1.73 ± 0.67). In addition, notable distinctions were detected in the Harris score between the groups at the Harris score 3 months postsurgery (*P* < .001) and at the last follow-up (*P* = .012). Furthermore, noteworthy distinctions were observed in the study group regarding both preoperative and postoperative leg length discrepancy (*P* < .001), in addition to preoperative offset and postoperative offset (*P* < .001). The utilization of the direct anterior approach in total hip replacement presents several advantages, including reduced tissue damage, decreased pain, quicker postoperative functional recovery, reduced dislocation risk, and enhanced hip joint functionality. This approach is in accordance with the tenets of minimally invasive surgery and improved recovery protocols, rendering it a feasible option for the management of developmental dysplasia of the hip among individuals.

## 1. Introduction

Developmental dysplasia of the hip (DDH) is a prevalent orthopedic ailment and a complex developmental disorder.^[1]^ It includes neonatal hip instability, acetabular dysplasia, and hip dislocation.^[2]^ DDH is a condition characterized by joint pain and dysfunction, in addition to a range of life-altering complications, including claudication, lordosis, scoliosis, and uneven lower limb lengths.^[3–5]^ The prevalence of DDH in China is 1.52%, with rates of approximately 0.75% among men and 2.07% among women, according to research.^[6]^ DDH is becoming more prevalent as the population ages,^[7]^ placing significant financial and societal strains on the provision of care and treatment. Although conservative and surgical approaches are available to treat DDH, the long-term effectiveness of conservative treatment is still uncertain, primarily because of the unpredictable progression of DDH to end-stage hip osteoarthritis.^[8]^ Due to protracted inadequate coverage and degeneration of the hip joint, adults with DDH are susceptible to developing progressive hip osteoarthritis; this condition frequently requires total hip arthroplasty (THA).^[9]^ Widespread and efficacious THA has been recognized as an intervention for severe hip joint disorders.^[10–12]^

In hip surgery, posterior THA is frequently employed, especially in elderly patients.^[13]^ The posterolateral approach (PLA) to THA has historically been preferred over the direct anterior approach (DAA) for patients with severe DDH.^[14–16]^ However, joint surgeons in China are becoming increasingly intrigued by the DAA.^[17]^ It is regarded as a minimally invasive alternative due to its ability to establish a natural path connecting nerve interfaces and muscle spaces. In comparison with other approaches, it is distinguished by diminished invasiveness, minimal damage to soft tissues, reduced bleeding, a diminished risk of dislocation, and an expedited recuperation period.^[18]^ Moreover, by enabling direct visualization and manual confirmation of the anatomical location of the acetabular cup, direct anterior THA is a dependable technique for performing effective surgery.^[19]^

The comparison of the 2 approaches holds significant value for joint surgeons in selecting surgical methods for treating congenital hip dysplasia. It also offers a practical foundation for addressing the ongoing debate regarding the preferred approach for surgical treatment of this condition. Comparing the treatment outcomes of patients with hip dysplasia who underwent direct anterior THA as opposed to PLA THA, this research group compared the 2 approaches from 2018 to 2023. The objective of the study was to evaluate the clinical effectiveness of both surgical approaches.

## 2. Methods

### 2.1. Patients

This is a retrospective study. The study incorporated a cohort of 201 patients who were diagnosed with DDH at the Trauma Center of the First Affiliated Hospital of Nanchang University between 2018 and 2023. The ethics code of this study is AF-SG-03-2.1-IIT. To protect the privacy of the participants, all personal information contained in the collected data was anonymized. This study complied with the ethical principles outlined in the Declaration of Helsinki and was granted clearance by the ethics committee of the hospital. Written consent from every patient was obtained.

### 2.2. Criteria for inclusion and exclusion

Enrollment in the research was contingent upon the following criteria being met: (1) adults with a diagnosis of DDH; (2) individual patients who failed conservative treatment and experienced progressive hip pain, claudication, and lower back pain due to DDH; (3) patients who underwent THA in our hospital between 2018 and 2023 using direct anterior and PLAs; (4) patients who have kept complete medical records and follow-up records for at least 6 months; (5) patients with complete medical records for comparison of the 2 surgical treatments; and (6) patients who have completed imaging studies. Patients who have the subsequent conditions are deemed ineligible: (1) active infection affecting the hip joint; (2) severe contracture or rigidity of the hip resulting from infection-related complications or prior surgical procedures; and (3) concurrent DDH and neurovascular disease, including paralysis from cerebral infarction, hemorrhage, and limb dysfunction from poliomyelitis.

### 2.3. Surgical technique

Every participant in the research was administered a combination of spinal and epidural anesthesia. On a conventional operating table, the study group was in a supine position, whereas the control group was in the lateral decubitus position. After disinfecting the surgical site, it was draped. In this retrospective study, patients were grouped based on the differences in their previously received surgical approaches. The choice of surgical approach was not influenced by any clear clinical indications, and there was no inherent preference or bias in the selection of the surgical approach. Surgeons presented both the DAA and PLA to patients, and the final decision was made based on the patient’s preference after receiving a detailed explanation of the options. Of the entire cohort of 201 patients, 100 underwent direct anterior THA, and 101 underwent THA via the posterior approach. The surgeries were performed by a team consisting of 2 chief physicians, 3 attending physicians, and 3 residents. However, all surgeries were led by the chief physicians, ensuring that the procedures adhered to strict standards. Both the DAA and PLA approaches were utilized by the surgeons, with the specific approach chosen based on patient preference and the surgeon’s expertise, in accordance with the hospital’s protocol.

### 2.4. Direct anterior approach total hip arthroplasty (study group)

**Figure 1.**
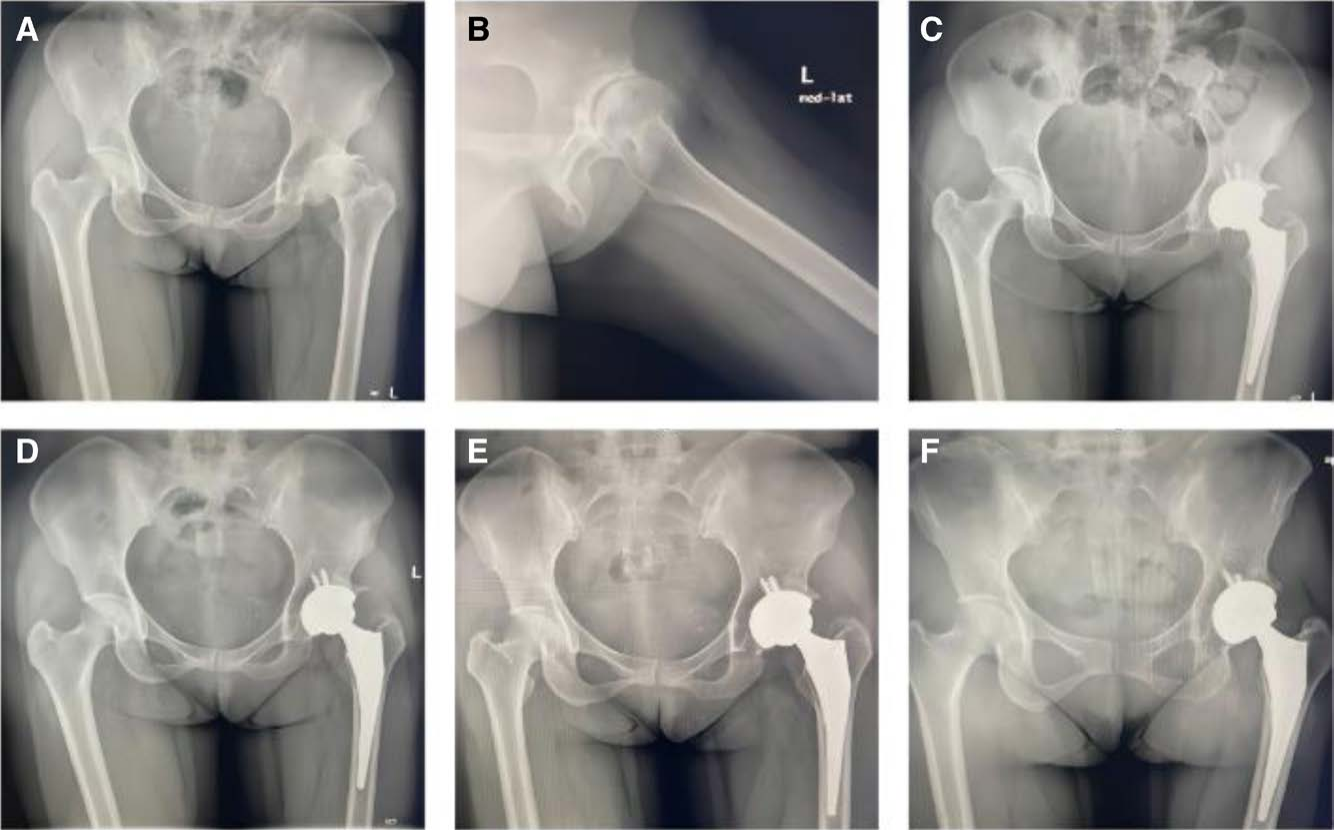
Direct anterior approach total hip arthroplasty for a 36-yr-old female patient. (A) Preoperative pelvis X-ray. (B) Preoperative hip X-ray. (C) Postoperative pelvis X-ray 1 wk after surgery. (D) Postoperative pelvic X-ray 3 mo after surgery. (E) Postoperative pelvic X-ray 6 mo after surgery. (F) Postoperative pelvic anteroposterior X-ray 1 yr after surgery.

A standard incision is performed in the muscle belly of the tensor fascia lata (TFL), commencing at a location 2 cm laterally to the anterior superior iliac spine and lasting for approximately 10-cm length. With great care, the skin, subcutaneous tissue, and superficial fascia are cut layer by layer with the intention of creating a wide incision. The superficial layer is dissected between the TFL and sartorius muscle, whereas the deep layer of fascia is dissected between the rectus femoris and vastus lateralis. By creating a T-shaped incision in the joint capsule, the femoral head and neck are exposed. The femoral neck is then cut with an oscillating saw approximately 0.5 to 1.0 cm above the lesser trochanter, and a bone knife is used to extract the femoral head in its entirety. Following adequate capsular release, osteophytes in close proximity to the acetabulum are meticulously eliminated, with particular attention paid to those located on the superior margin of the acetabulum. Using an acetabular file, the surface of the acetabular joint is prepared, beginning with a small size and maintaining an abduction angle of 45° and an anteversion direction of 20° until blood seeping is evident and cancellous bone is visible. Subsequent to the sizing of a trial mold, a suitable biological acetabular cup prosthesis is selected, implanted, and secured with 2 screws. Using a medullary cavity file, the femoral side medullary cavity is exposed; the file progressively enlarges as the file size increases. A biological femoral stem prosthesis is chosen in conjunction with size testing and an appropriate femoral head. The hip joint is examined for containment and satisfactory range of motion in all directions following reduction. After performing a thorough flush of the wound with saline and hydrogen peroxide, a drainage tube is inserted, and the incision is sealed through layer-by-layer suturing of the musculature, fascia lata, and subcutaneous fat. For the visual representation, refer to Figure 1.

### 2.5. Posterolateral approach total hip arthroplasty (control group)

**Figure 2.**
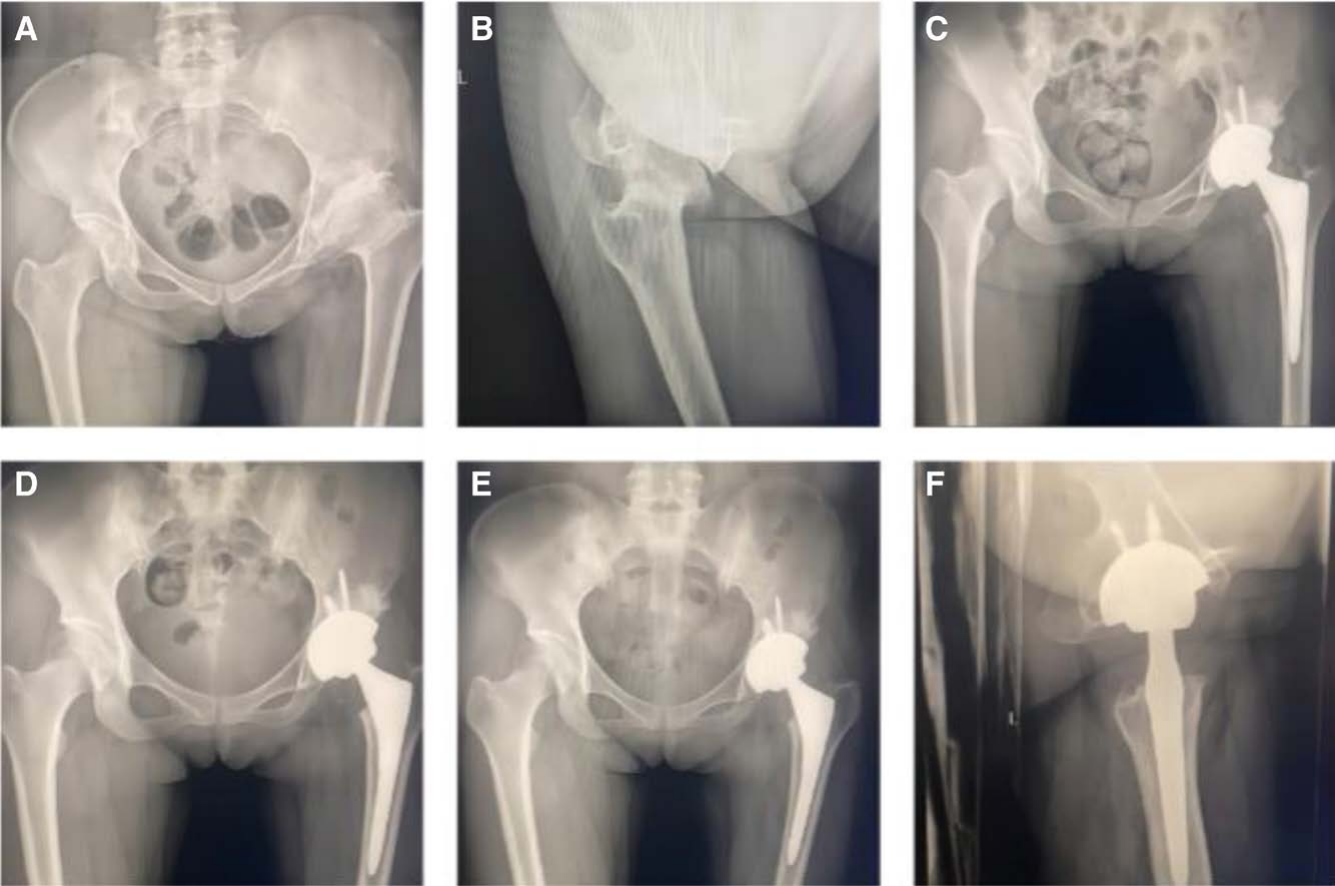
Posterolateral approach total hip arthroplasty for a 37-yr-old female patient. (A) Preoperative pelvis X-ray. (B) Preoperative hip X-ray. (C) Postoperative pelvis X-ray 3 mo after surgery. (D) Postoperative pelvic X-ray 6 mo after surgery. (E) Postoperative pelvic X-ray 1 yr after surgery. (F) Postoperative hip X-ray 1 yr after surgery.

On the affected side, an approximate 12-cm-long posterolateral incision is made around the body surface trace of the greater trochanter. After making a layer-by-layer incision through the skin, subcutaneous tissue, and superficial fascia, the fascia lata is cut. To expose the hip joint on the affected side, the femoral insertion points of the gluteus maximus muscle, the piriformis muscle, the superior and inferior gemellus muscles, and the obturator internus muscles at their attachment points are subsequently cut. The femoral neck is cut at a distance of 1.5 cm above the lesser trochanter using an oscillating saw. Subsequently, the femoral head is entirely extracted using an osteotome. Following the removal of the hyperplastic joint capsule surrounding the hip, the hip articular surface is ground in a sequential manner utilizing a hip scraper until a slight amount of bleeding results from the exposed cancellous bone. Implantation of a minute quantity of cancellous bone. After selecting and implanting the suitable biological acetabular cup prosthesis, it is secured with screws. Utilizing a medullary cavity file, the femoral medullary cavity is initially exposed, after which the cavity is subsequently expanded in a sequential fashion. On the basis of size testing, an appropriate femoral stem prosthesis and femoral head are chosen. The iliotibial band and rectus femoris muscle are released before reduction. After resetting the prosthesis, appropriate hip flexion and extension are restored. The hip joint is examined for containment and satisfactory range of motion in all directions following reduction. As illustrated in Figure 2, a drainage catheter is subsequently inserted beneath the muscle layer, and the incision is progressively closed in sections.

### 2.6. Preoperative treatment

All patients were subjected to preoperative pelvic imaging, anticoagulation with low molecular weight heparin, fluid rehydration and anti-infective therapy 30 minutes before the procedure, and a 12-hour period with restricted water consumption before the operation.

### 2.7. Postoperative treatment

X-rays of the pelvis were performed on every patient the day after the operation. After surgery, the drainage catheter will be extracted between 24 and 72 hours later, contingent upon the condition of the drainage fluid. Incision dressings must be changed every 2–3 days. Antibiotic prescriptions are contingent upon the dimensions of the incision, whereas analgesics are contingent upon the patient’s pain tolerance. Heparin with a low molecular weight was administered 6 hours after surgery for anticoagulation purposes. After surgery, patients were instructed to take rivaroxaban orally for a minimum of 4 weeks and up to a maximum of 5 weeks following their discharge. Rivaroxaban would only be discontinued early if there were specific medical indications, such as significant bleeding complications or other contraindications to continued anticoagulation therapy. To ensure patients did not stop taking rivaroxaban prematurely, close follow-up and monitoring were implemented to confirm adherence and evaluate the ongoing need for anticoagulation. Patients were instructed to report any adverse symptoms promptly, and their anticoagulation therapy was adjusted based on their clinical condition. It was recommended that patients begin kicking exercises beside their beds on the 3rd day following the operation and should advance to walking with crutches 1 week later.

### 2.8. Follow-up and outcome evaluation

Patients were regularly followed up on a routine basis at 3 months, 6 months, and 1 year following the procedure. A visual analog scale (VAS) was employed to evaluate the intensity of postoperative pain 1 day, 3 days, and 1 week following the procedure. The Harris score^[20]^ was employed to assess the functional rehabilitation of the hip joint following surgery. The measurement of leg length discrepancy (LLD) involved the drawing of a line connecting 2 teardrops bilaterally, with the perpendicular line to the lesser trochanter being measured. LLD was denoted by the absolute value of the bilateral distance difference. From the lower edge of the teardrop to the long axis of the femoral shaft, the femoral offset was measured. To determine the absolute value of the discrepancy in bilateral offsets, the offset difference (-d) was utilized. Surgical incision length, intraoperative blood loss, blood transfusion volume, operation time, hospitalization time, preoperative and postoperative LLD, and femoral offset difference were among the parameters that were recorded. Following surgical procedures, patients underwent routine follow-up evaluations at the orthopedics department. These evaluations primarily aimed to assess the weight-bearing capacity of the afflicted limb and monitor hip mobility. Physical examinations and pelvic X-rays were performed at each follow-up appointment to detect potential complications, including prosthetic dislocation or infection. The Harris^[21]^and Barthel index^[22]^ were utilized to evaluate the functional recovery and activities of daily living of patients at the final postoperative follow-up.

### 2.9. Statistical analysis

Statistical analysis was performed on the data using SPSS 27.0.1 (IBM Co., Armonk). A *P*-value below .05 was deemed to indicate statistical significance. The units of measurement were expressed in x ± s. For measurement data, the independent sample *t* test was applied; for count data, the chi-square test or Fisher’s exact test was utilized.

## 3. Results

### 3.1. General information

Regarding preoperative demographic information, no statistically significant difference existed between the 2 groups (*P* > .05). For more details, please refer to Table 1.

**Table 1:** General information.

Indicator	Group		*t*	*P*
	Control group (n = 101)	Study group (n = 100)		
Age (yr)	57.77 ± 11.29	57.04 ± 9.94	0.24	.811
Female	60	64	-	.570
Male	41	36		
BMI (kg/m²)	22.85 ± 4.95	23.97 ± 2.79	−0.967	.338
Symptomatic history (yr)	10.14 ± 11.48	7.33 ± 6.79	1.039	.304

BMI = body mass index.

#### 3.1.1. Comparison of intraoperative and postoperative indicators

The average intraoperative blood loss for the study group was 545.83 ± 169.34 mL, which was considerably greater than the control group’s 215.39 ± 65.98 mL (*P* < .001). The operation time of 137.71 ± 41.18 minutes for the study group was marginally longer than that of 127.27 ± 44.09 minutes for the control group (*P* = .392). The duration of hospitalization for the study group was 11.29 ± 2.39 days, whereas for the control group, it was 12.81 ± 3.02 days (*P* = .056). In the study group, the volume of blood transfusion was 47.92 ± 142.55 mL, whereas in the control group, it was 112.31 ± 189.57 mL (*P* = .184). The length of the surgical incisions in the study group was 10.42 ± 1.18 cm, whereas in the control group, it was 13.15 ± 1.35 cm (*P* < .001). Although there were no significant variations in operation time, hospitalization time, or blood transfusion volume among the groups, there were noteworthy distinctions in the length of surgical incisions and intraoperative blood loss. Refer to Table 2 for more details.

**Table 2:** Comparison of intraoperative and postoperative indicators between the 2 groups.

Indicator	Group		*t*	*P*
	Control group (n = 101)	Study group (n = 100)		
Preoperative VAS score	2.12 ± 0.33	1.67 ± 0.64	3.17	.003
VAS score 1 d after surgery	7.46 ± 0.51	6.71 ± 0.46	5.46	<.001
VAS score 3 d after surgery	6.35 ± 0.49	5.71 ± 0.46	4.74	<.001
VAS score 1 wk after surgery	1.73 ± 0.67	0.96 ± 0.20	5.44	<.001
Intraoperative blood loss (mL)	215.39 ± 65.98	545.83 ± 169.34	−9.226	<.001
Operative time (min)	127.27 ± 44.09	137.71 ± 41.18	−0.86	.392
Hospital stay (d)	12.81 ± 3.02	11.29 ± 2.39	1.96	.056
Length (cm)	13.15 ± 1.35	10.42 ± 1.18	7.62	<.001
Blood transfusion volume (mL)	112.31 ± 189.57	47.92 ± 142.55	1.35	.184

#### 3.1.2. Comparison of preoperative and postoperative pain scores

The average preoperative VAS score for the study group was 1.67 ± 0.64, whereas the average preoperative VAS score for the control group was 2.12 ± 0.33 (*P* = .003). The patients in the study group exhibited VAS scores (6.71 ± 0.46, 5.71 ± 0.46, 0.96 ± 0.20) 1 day, 3 days, and 1 week following surgery, respectively, in comparison with the control group (7.46 ± 0.51, 6.35 ± 0.49, 1.73 ± 0.67). There is a significant difference (*P* < .001). Refer to Table 3.

**Table 3:** Comparison of functional scores and activities of daily living between the 2 groups.

Indicator	Group		*t*	*P*
	Control group (n = 101)	Study group (n = 100)		
Follow-up time (yr)	1.78 ± 0.53	1.95 ± 1.13	−0.70	.488
Harris, preoperative	40.08 ± 6.46	35.83 ± 6.20	2.37	.022
Harris, 3 mo	84.35 ± 3.43	88.67 ± 2.79	−4.86	<.001
Harris, last follow-up	89.62 ± 4.25	92.38 ± 3.06	−2.61	.012
Barthel index, preoperative	92.31 ± 10.88	93.33 ± 13.49	−0.30	.768
Barthel index, 3 mo	94.31 ± 9.27	94.67 ± 11.75	−0.12	.905
Barthel index, last follow-up	95.92 ± 8.40	96.00 ± 10.30	−0.30	.977

#### 3.1.3. Comparison of functional scores and activities of daily living ability

The mean follow-up time for the study group was 1.95 ± 1.13 years, whereas it was 1.78 ± 0.53 years for the control group (*P* = .488). The preoperative Harris score (*P* = .022) and Barthel index (*P* = .768) did not differ significantly between the 2 groups. The Harris scores for the study group and the control group were 88.67 ± 2.79 and 84.35 ± 3.43, respectively, 3 months after the procedure (*P* < .001). The final follow-up assessment revealed that the Harris score for the study group was 92.38 ± 3.06, while for the control group, it was 89.62 ± 4.25 (*P* = .012). Significant disparities in Harris scores were observed between the groups during the final follow-up and 3 months after the procedure. Three months after the procedure, the Barthel index for the study group was 94.67 ± 11.75, whereas it was 94.31 ± 9.27 for the control group (*P* = .905). At the final follow-up, the Barthel index for the study group was 96.00 ± 10.30, whereas it was 95.92 ± 8.40 for the control group (*P* = .977). For more details, please refer to Table 3.

### 3.2. Radiological data

The preoperative LLD for the study group was 12.33 ± 7.48, whereas for the control group, it was 15.37 ± 15.17 (*P* = .381). Following the operation, the postoperative LLD for the study group was 4.75 ± 3.14, whereas it was 6.09 ± 3.65 for the control group (*P* = .174). The preoperative offset was 7.27 ± 4.41 in the study group and 8.05 ± 3.66 in the control group (*P* = .500). In the study group, the postoperative deviation was 3.96 ± 2.62, whereas it was 3.84 ± 2.19 in the control group (*P* = .866). No substantial disparities were observed in LLD and Offset-D values between the 2 groups. Specifically, the preoperative LLD for the study group was 12.33 ± 7.48; it decreased to 4.75 ± 3.14 (*P* < .001) following the operation; the preoperative offset was 7.27 ± 4.41, and it improved to 3.96 ± 2.62 (*P* < .001). The preoperative LLD for the control group was 15.37 ± 15.17, and it decreased to 6.09 ± 3.65 (*P* = .001); the preoperative offset was 8.05 ± 3.66, and it decreased to 3.84 ± 2.19 (*P* < .001) in the postoperative period. In both groups, LLD and offset exhibited substantial improvement. Refer to Table 4 for more details.

**Table 4:** Comparison of radiological data variation results between the 2 groups.

Group	LLD (mm)				Offset-D (mm)			
	Preoperative	Postoperative	*t*	*P*	Preoperative	Postoperative	*t*	*P*
Study	12.33 ± 7.48	4.75 ± 3.14	7.36	<.001	7.27 ± 4.41	3.96 ± 2.62	6.56	<.001
Control	15.37 ± 15.17	6.09 ± 3.65	3.59	.001	8.05 ± 3.66	3.84 ± 2.19	10.46	<.001
*t*	0.89	1.38	-	-	0.68	−0.17	-	-
*P*	.381	.174	-	-	.500	.866	-	-

### 3.3. Postoperative complications

**Figure 3.**
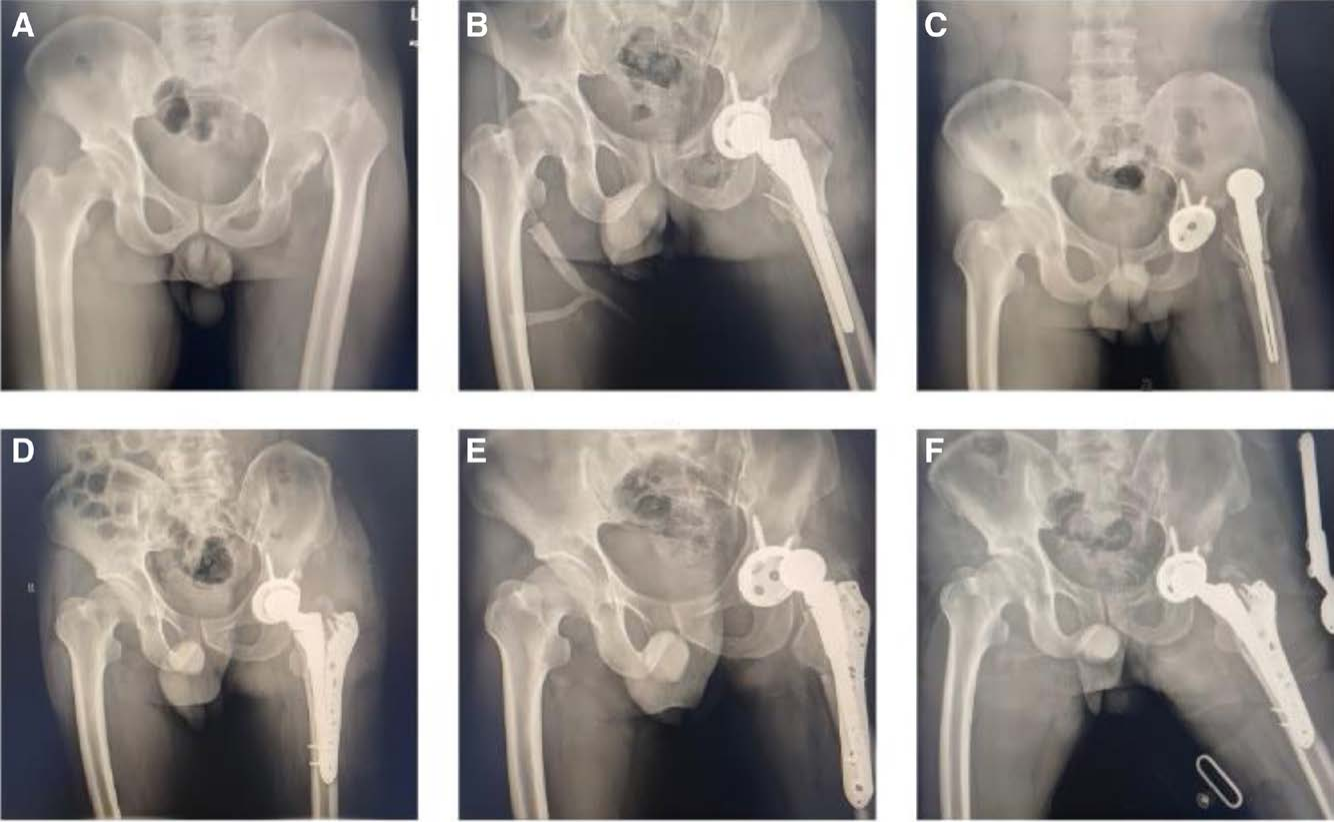
Prosthetic dislocation combined with surrounding fractures for a 48-yr-old male patient. (A) Preoperative pelvis X-ray. (B) Postoperative pelvis X-ray. (C) Anterior dislocation combined with surrounding fractures after surgery. (D) Pelvic X-ray 3 mo after revision surgery. (E) Anterior dislocation after revision surgery. (F) Pelvic X-ray after manual reduction surgery.

**Figure 4.**
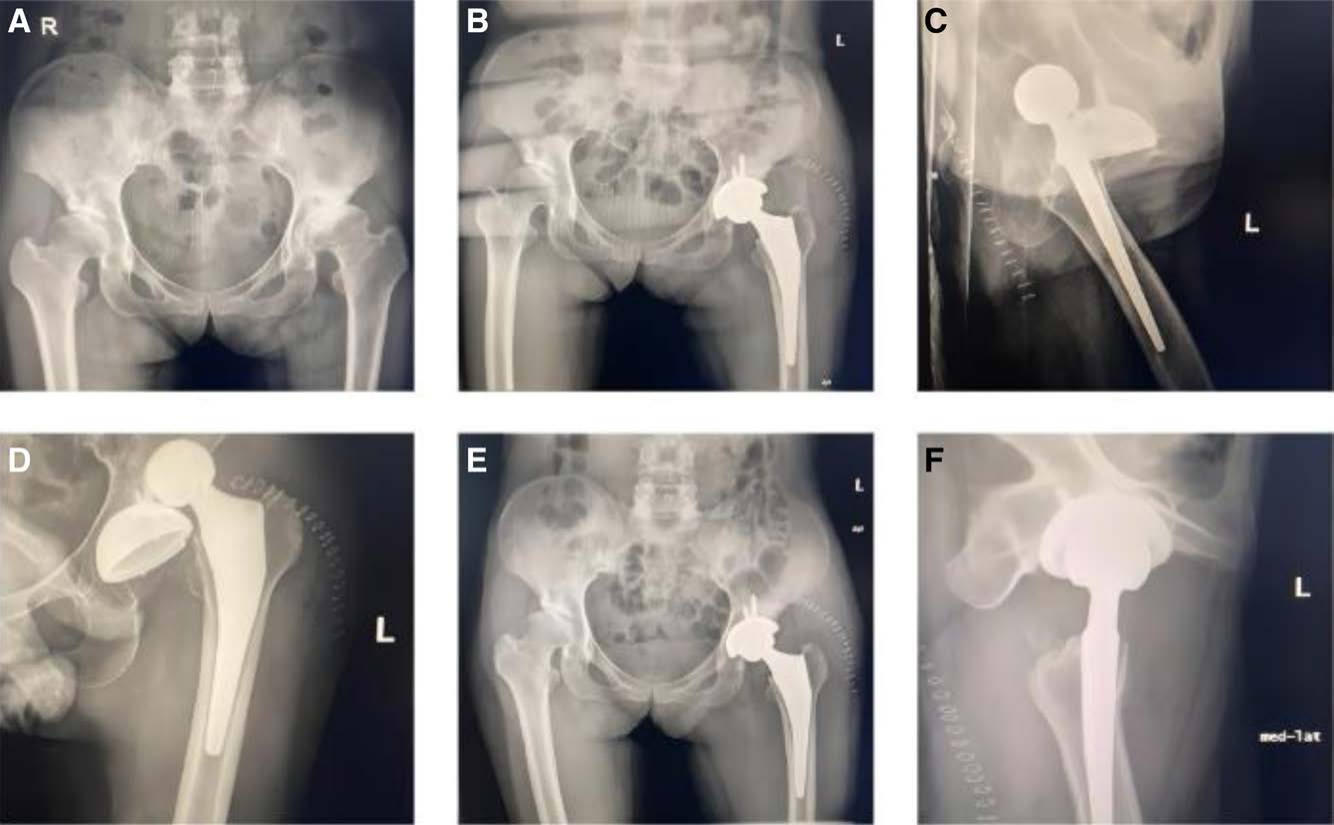
Prosthetic dislocation for a 56-yr-old female patient. (A) Preoperative pelvis X-ray. (B) Preoperative pelvis X-ray. (C, D) Anterior dislocation hip X-ray after surgery. (E) Pelvic X-ray after manual reduction surgery. (F) Hip X-ray after manual reduction surgery.

No postoperative complications were identified in the patients comprising the study group throughout the follow-up period. Four patients in the control group, in contrast, encountered complications following surgery. As detailed in Table 5, these included 3 instances of postoperative prosthetic dislocation (*P* = .086), all of which were cases of posterior dislocation, and one instance of inadequate incision healing (*P* = .332). One member of the patients in the control group experienced an unintentional fall after being discharged, which resulted in the dislocation of a prosthetic device and subsequent fractures, which required revision surgery. Following surgery, the prosthesis dislodged once more despite manual reconstruction, necessitating additional manual reduction, as illustrated in Figure 3. As illustrated in Figure 4, manual reduction successfully mitigated the remaining 3 instances of prosthetic dislocation. Furthermore, 1 patient presented with suboptimal wound healing, which was evidenced by enduring redness, swelling, and exudation. Antibiotics, anti-infection agents, and, if required, debridement and exploratory surgery comprised the treatment regimen. Infection prevention, fluid rehydration, and analgesia were administered as postoperative care.

**Table 5:** Comparison of postoperative complications between the 2 groups.

Group	n	Postoperative prosthetic dislocation	Inadequate incision healing
Study	101	0	0
Control	100	3	1
*P*	-	.086	.332

## 4. Discussion

Hip dysplasia is a substantial hip disorder that may be managed via a range of surgical interventions, including pelvic osteotomy, femoral osteotomy, hip fusion, and total hip replacement. At present, the treatment regarded as the most efficacious approach for hip joint dysplasia is total hip replacement.^[22]^ Numerous physicians have investigated and implemented various surgical techniques subsequent to the clinical progression of hip replacement. These techniques include the DAA, anterolateral approach, posterior approach, and PLA.^[23–26]^ Currently, the PLA is the prevailing method due to its simplicity and abbreviated duration of operation.^[27]^ The indications for posterior lateral approach THA are extensive. However, this methodology entails the severance of internal and external obturator muscles, piriformis muscles, and the incision of the posterior joint capsule. As a consequence, substantial damage is inflicted upon soft tissues, patients endure postoperative pain, and there is an elevated likelihood of hip joint dislocation,^[28]^ which may hinder the recovery of joint function after surgery. The DAA has gained significant popularity among bone and joint surgeons worldwide due to advancements in minimally invasive techniques and increases in living standards. The methodology referred to as DDA has garnered considerable popularity in the United States and select European nations.^[29,30]^ The survey indicates that the aggregate quantity of DAA in China has multiplied by 3 in 2021 compared with 2015.^[31]^ The DAA, which was initially introduced by American scholar Hueter in 1881, became increasingly well-known subsequent to its implementation by physicians such as Smith-Petersen in 1917.^[32]^ This methodology, which is based on the Smith-Petersen technique, entails penetrating via the intermuscular compartment bounded by the rectus femoris, sartorius, and TFL.^[33]^ By bringing the incision closer to the exterior, it is unnecessary to make an incision through the pertinent muscle groups that envelop the joint. The operation is performed at the interface of the neurovascular and intermuscular systems, which minimizes injury to soft tissues, expedites functional recovery after surgery, and produces favorable results.

Comparing the clinical outcomes of direct anterior THA and PLA THA for the treatment of DDH, this study validated the efficacy of both surgical methods. A comparison was made between the 2 groups regarding the extent of soft tissue injury through the observation of the length of the incision and intraoperative blood loss. In the group that received the PLA, the incision length was considerably greater than in the group that received the direct approach. Consequently, the DAA resulted in reduced injury and trauma compared with the PLA. However, it is worth noting that the intraoperative blood loss was considerably greater in the direct approach group as opposed to the PLA group. This was primarily attributable to the lengthy learning curve, complexity, extended duration, and ultimately resulting in higher intraoperative blood loss associated with the DAA in comparison with the PLA. Consequently, joint surgeons must possess expertise in the direct approach technique. It is advisable to address the principal branch of the ascending branch of the lateral femoral circumflex artery promptly when separating the TFL and sartorius muscles. This will contribute to a further reduction in intraoperative blood loss. Concurrently, the ascending branch of the lateral circumflex femoral artery should not be approached excessively closely to the TFL muscle’s entrance site when manipulating its branches. This particular region is susceptible to potential damage to its nerve supply.^[34]^ In their retrospective analysis, Li et al^[35]^ compared the clinical outcomes of total hip replacements performed using the DAA versus the PLA. A notable disparity in intraoperative blood loss was identified between the groups that underwent direct anterior and PLAs. In addition, the DAA group exhibited a shorter incision length. Consistent with prior research, these results suggest that total hip replacement via DAA might entail a lesser degree of invasiveness than that performed via PLA.

The DAA offers several benefits, including expedited restoration of muscle strength, joint activity, gait, and other functions, as well as a reduced incidence of postoperative pain.^[36,37]^ The findings of this study indicate that the DAA to total hip replacement yields superior functional recovery, reduced postoperative pain scores, and higher Harris scores in patients compared with the PLA. The preservation of muscle tissue during the surgical procedure and the subsequent enhancement of joint stability account for this outcome. The DAA preserves the structural integrity of the posterior muscle and soft tissue while repairing the anterior joint capsule via Hueter’s anatomical breach without causing lateral muscle amputation. As a result, individuals undergo postoperative pain alleviation, artificial joint stability is improved, they are able to immediately commence a range of rehabilitation exercises, and they attain functional recovery at an accelerated pace. During early rehabilitation, the DAA results in a quicker recovery of hip function and less discomfort, according to Parvizi et al. The DAA group experienced speedier recovery of joint function and milder postoperative pain symptoms, according to the research of Rodriguez et al.^[38]^ In the same vein, the Harris score for the DAA was found to be considerably higher than that of the lateral approach 6 weeks after surgery, according to research by Berend et al.^[39]^ These results are consistent with those of the present study and indicate that a DAA to total hip replacement is beneficial for functional rehabilitation in the early postoperative period.

In comparison with preoperative measurements, radiographic analysis demonstrated a substantial increase in the LLD and femoral offset difference. These enhancements are of the utmost importance in reinstating biomechanical alignment and stabilizing joints. Prior research suggests that individuals may perceive a distinction in LLD ranging from 5 to 10 mm; this can be compensated for through adjustments to the spine and pelvis. Nevertheless, the body’s compensatory mechanisms might become overwhelmed when LLD surpasses 10 mm.^[40]^ The correlation between femoral deviation and acetabular wear was underscored by Little et al,^[41]^ who also emphasized that deviations exceeding 5 mm have the potential to hasten wear. In addition, improved clinical outcomes are associated with the combined effects of leg length and offset after THA.^[42]^

A prevalent complication of total hip replacement is dislocation.^[43]^ Dislocations in postoperative patients are frequently brought on by actions including squatting, hip flexion, adduction, internal rotation, or falling. Kwon et al reported that the posterior approach resulted in no complications, with a dislocation rate of 4.46%. In their analysis of 494 primary total hip replacement cases utilizing the DAA, Matta et al^[44]^ identified a dislocation rate of 0.6% postoperatively. Postoperative prosthesis dislocation was observed in 3 patients assigned to the PLA group in our study. Conversely, the DAA group did not experience any dislocations, for a cumulative rate of 6%. This implies that employing the DAA as opposed to the PLA might mitigate the likelihood of dislocation.

Recent studies have clearly shown that the DAA not only has significant advantages in terms of surgical time and postoperative recovery but also reduces the occurrence of postoperative complications. However, this does not mean that DAA is suitable for all patients. In particular, for patients with certain anatomical structures or special conditions, the PLA still demonstrates its unique value. For example, DAA, due to its smaller incision and protection of important structures, can effectively reduce intraoperative blood loss and accelerate postoperative recovery. However, it has a higher technical difficulty and requires a longer learning curve, with improper early-stage techniques potentially leading to repeated fluoroscopy and longer surgical time. Moreover, DAA may cause significant damage to key muscles, particularly the gluteus medius and tensor fasciae latae, which can affect postoperative functional recovery. As a result, in inexperienced hands, the complication rate may rise, and additional incisions may be needed. On the other hand, while the PLA involves more extensive muscle dissection, it is a relatively familiar procedure with a shorter learning curve and remains suitable for high-risk patients, such as those with obesity or hip dysplasia. For complex hip joint pathologies, especially those involving severe deformities or joint fixation, PLA provides better exposure and more established technical approaches. Furthermore, the indications and limitations of the DAA should be further explored. While DAA offers good surgical outcomes for most patients, its applicability is limited for certain patients with specific clinical backgrounds, such as severe obesity or congenital hip dysplasia (e.g., Crowe type 3). In these cases, PLA or other traditional approaches may be more appropriate. In summary, both DAA and PLA have their own advantages and disadvantages. When choosing the appropriate surgical approach, individual patient conditions must be comprehensively considered. While DAA offers clear benefits in terms of reducing surgical trauma and accelerating recovery, its longer learning curve and reliance on the surgeon’s experience mean that early users may face more complications. Therefore, further research and comparative analysis will be helpful in understanding the advantages of both approaches and providing personalized treatment options for different patients.

There are certain limitations to this study. First, it is imperative to acknowledge that the analysis was conducted retrospectively, which may have introduced selection bias. Furthermore, the presented data are constrained in scope due to its origin in a solitary center and the relatively small number of direct anterior total hip arthroplasties. Further research should prioritize the enlargement of sample sizes and the extension of follow-up periods to facilitate more reliable comparisons. Hence, it is recommended that future research endeavors give precedence to prospective randomized controlled trials that incorporate larger sample sizes.

## 5. Conclusion

The utilization of the DAA in total hip replacement presents numerous advantages, including reduced tissue damage, decreased pain, quicker postoperative functional recovery, a lower risk of dislocation, and improved hip joint function. This methodology is in accordance with the tenets of minimally invasive surgery and enhanced recovery protocols, rendering it a feasible option for the management of DDH among individuals.

## Author contributions

**Conceptualization:** Wuyuanhao Lin.

**Data curation:** Wuyuanhao Lin.

**Formal analysis:** Wuyuanhao Lin.

**Funding acquisition:** Wuyuanhao Lin.

**Investigation:** Wuyuanhao Lin.

**Validation:** Wuyuanhao Lin.

**Visualization:** Wuyuanhao Lin.

**Writing—original draft:** Wuyuanhao Lin.

**Writing—review & editing:** Wuyuanhao Lin.
